# Efficient multitool/multiplex gene engineering with TALE-BE

**DOI:** 10.3389/fbioe.2022.1033669

**Published:** 2022-11-10

**Authors:** Alex Boyne, Ming Yang, Sylvain Pulicani, Maria Feola, Diane Tkach, Robert Hong, Aymeric Duclert, Philippe Duchateau, Alexandre Juillerat

**Affiliations:** ^1^ Cellectis Inc., New York, NY, United States; ^2^ Cellectis, Paris, France

**Keywords:** gene editing, base editors, TALE, t-cells, cell engineering

## Abstract

TALE base editors are a recent addition to the genome editing toolbox. These molecular tools are fusions of a transcription activator-like effector domain (TALE), split-DddA deaminase halves, and an uracil glycosylase inhibitor (UGI) that have the distinct ability to directly edit double strand DNA, converting a cytosine (C) to a thymine (T). To dissect the editing rules of TALE-BE, we combined the screening of dozens of TALE-BE targeting nuclear genomic loci with a medium/high throughput strategy based on precise knock-in of TALE-BE target site collections into the cell genome. This latter approach allowed us to gain in depth insight of the editing rules *in cellulo*, while excluding confounding factors such as epigenetic and microenvironmental differences among different genomic loci. Using the knowledge gained, we designed TALE-BE targeting CD52 and achieved very high frequency of gene knock-out (up to 80% of phenotypic CD52 knock out). We further demonstrated that TALE-BE generate only insignificant levels of Indels and byproducts. Finally, we combined two molecular tools, a TALE-BE and a TALEN, for multiplex genome engineering, generating high levels of double gene knock-out (∼75%) without creation of translocations between the two targeted sites.

## Introduction

Base editing, one of the most recent advances in the field of genome editing is a technology that allows the introduction of point mutations (transitions C>T or A>G and cytosine transversion C>G) in defined loci of a targeted DNA sequence (Koblan et al., 2018; Anzalone et al., 2020; Kurt et al., 2021; Zhao et al., 2021). Base editors create mutations by deamination at the targeted bases (C or A), which are subsequently converted into T or G during the DNA repair process. Such a process does not create DNA double strand breaks as does CRISPR/Cas9, or other engineered nucleases, and is a promising therapeutic strategy for genetic diseases.

In contrast to the Cas9 and Cpf1 base editing platforms that operate predominantly on ssDNA, a newly discovered bacterial deaminase (DddA) catalyzes the deamination of cytidine within double strand DNA molecules and allows for the development of designer base editors with alternative DNA targeting platforms (Mok et al., 2020). These TALE-BE, and very recently Zinc finger BE (Lim et al., 2022), were used for several applications including the creation of mutations in nuclear DNA (Mok et al., 2020), and, unlike Cas9 and Cpf1 base editors, also mitochondrial DNA (Lee et al., 2021; Qi et al., 2021; Sabharwal et al., 2021; Guo et al., 2022; Liu et al., 2022; Wei et al., 2022), or chloroplast (Kang et al., 2021; Nakazato et al., 2021), generating inheritable modifications and rendering the TALE-BE the first functional base editing tool available for these latter cellular compartments. Indeed, these new TALE-BE expand the base editing toolbox, providing additional ways to target specific sites for correction. For instance TALE-BE, like other TALE-derived editors, should bring with them enhanced ability to access hard to edit loci (Jain et al., 2021). And by utilizing different rules for targeting, and interacting with, the genome, these editors will open up additional sites outside of the scope of previously described Cas9 and Cpf1 base editing platforms. However, despite these successful applications, more detailed and comprehensive study are necessary to fully promote the development of these new molecular tools. In particular, while it also requires high modification frequencies to ensure compatibility with product development, multiplex gene editing using base editors represents a promising strategy to avoid unintended translocations between edited loci.

Here, we studied the determinants to achieve high editing frequencies using TALE-BE. We combined the screening of several TALE-BE targeting various endogenous loci with the development of a medium/high throughput cell-based assay that would avoid biases due to confounding effects such as epigenomic factors or modifications (Valton et al., 2012; Bose et al., 2021). The accumulated knowledge enabled the definition of TALE-BE design guidelines that were further applied to nuclear base editing, allowing for very efficient knock-out of CD52 in primary T-cells (up to 87% phenotypically and 86% editing at the genomic level). We further demonstrated the possibility of combining a TALEN (TALE nuclease) and a TALE-BE (TALE Base editor) to perform a double gene KO of *TRAC* and *CD52* (75% double negative cell population), a combination of target genes used for allogeneic CAR T-cell adoptive therapies. Such combinations of molecular tools open the way to simultaneous multiplex gene engineering with more controllable outcomes.

## Results

### Comparison of TALEN and TALE-BE efficiencies on nuclear genomic loci

**FIGURE 1:**
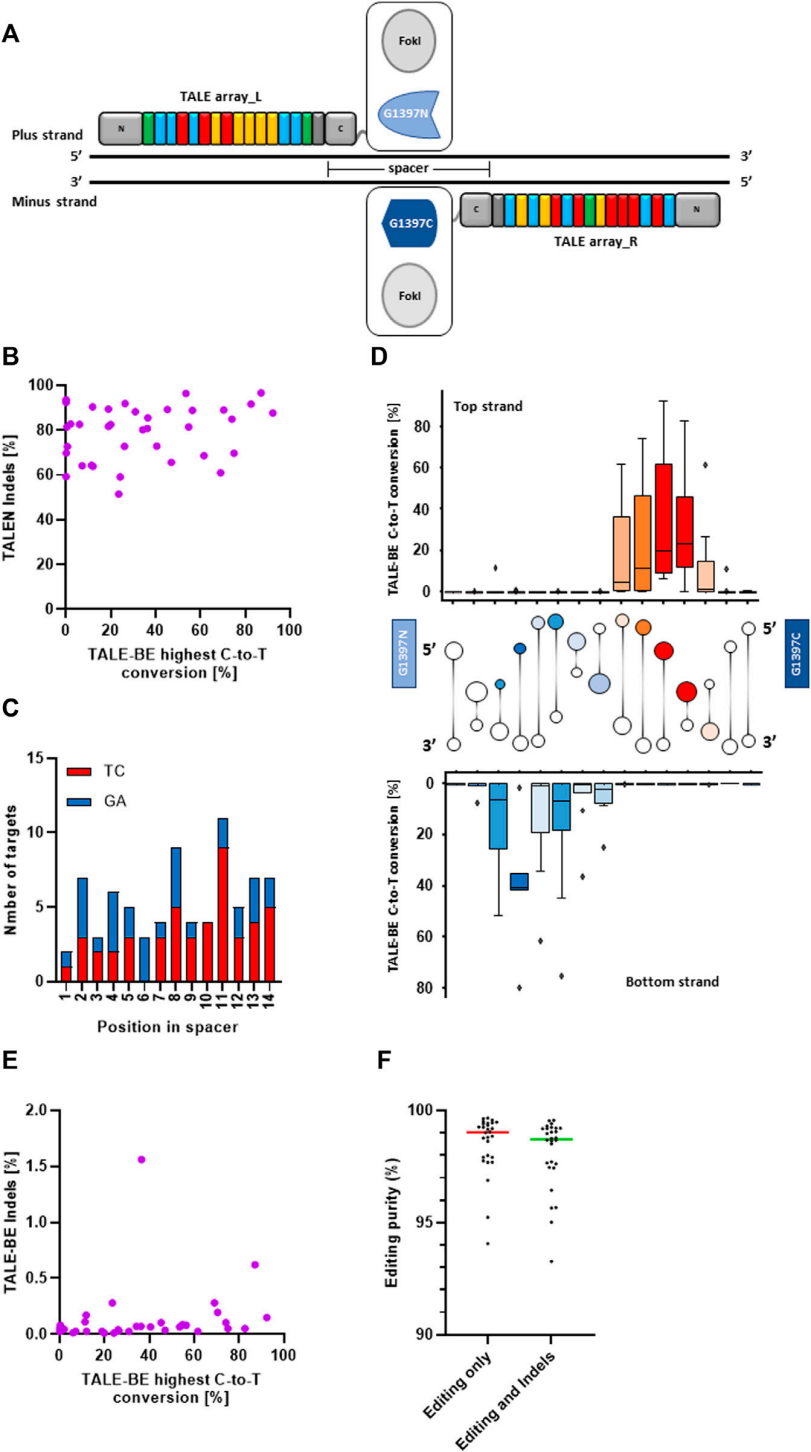
**(A).** Schematic representation of a TALEN and a TALE-BE. **(B).** Average of the highest C-to-T conversion frequencies of 37 base editors (among all edited bases within the target spacer region) versus the frequencies of indels created by the matching TALEN (*N* = 2, independent T-cells donors). **(C).** Repartition of TC/GA in the spacer of the 37 TALE-BE. **(D).** C-to-T conversion frequencies of the target C (top) or G (bottom) at different positions within the 15 bp TALE-BE spacer (box represent 5%–95% percentile, whiskers represent min and max) with cartoon of DNA double helix drawn between top and bottom strands to aid visualization of edited positions within the spacer. Colors of cartoon are selected to match those of the box plot, with darker colors indicating more editing. **(E).** Average of the highest C-to-T conversion frequencies of 37 base editors versus indels frequencies generated within the spacer. **(F).** Editing purity (median) within the cell population. Left: C-to-T conversion within the C-to-A/G/T population. Right: C-to-T conversion within the C-to-A/G/T + Indels population. For all panels: *N* = 2, independent T-cells donors.

To consolidate our understanding of the determinants for efficient editing by TALE-BE, we first identified a set of 37 TALEN (TALE nucleases, Figure 1A), originating from a previously described backbone (Valton et al., 2012; Juillerat et al., 2014; Gautron et al., 2017), that showed high activity in primary T-cells (median indels = 82% and s.d. = 12) (Figure 1A, Supplementary Figure S1A). These 37 target sequences (Supplementary Table S1) were also carefully selected to target regions with different chromatin states in T cells as found by ChromHMM (Ernst and Kellis 2017, Supplementary Table S1). In addition, the spacer sequence, the DNA sequence between the two TALE binding sites, was also kept constant to 15 bp as it was previously shown to accommodate both TALEN and TALE-BE (Juillerat et al., 2014; Mok et al., 2020). Finally, the sequence of the spacers contained various numbers of, but homogeneously distributed, Cs, Gs, TCs or GAs as previous studies demonstrated a strong editing preference for 5′-TC contexts (Figure 1C; Supplemntary Figures S1B,C) (Mok et al., 2020). We then produced the corresponding 37 TALE-BE by replacing the FokI catalytic domain with the DddAtox split and an uracil glycosylase inhibitor (UGI). We focused on the so-called G1397 split, in which the deaminase is split at the C-terminus of G1397 residue, as this fusion previously showed improved editing activity (Mok et al., 2020) (Figure 1A). We then compared, in primary T-cells, the maximum editing within the spacer for a given TALE-BE to the Indel frequencies created by the corresponding TALEN counterpart (Figure 1B). The lack of correlation (Spearman correlation = 0.16, *p*-value = 0.33) between the two data sets (TALEN vs. TALE-BE editing frequencies) confirms that the key determinant for efficient editing is related to the positioning of the target cytosine within the spacer. Indeed, analysis of editing efficiency in function of the position within the spacer highlighted a defined 4–5 bp editing window on both, top and bottom strands, occupying approximately the same three dimensional space when visualized along the DNA double helix (Mok et al., 2022) (Figure 1D).

Interestingly, low frequencies of Indels (small insertion and deletions, <0.5%) were observed for 35 out of 37 base editors (Indel frequencies: median = 0.06% and s.d. = 0.17, Figure 1E). The Indels observed at the target site moderately correlated with editing frequency within the spacers (Spearman correlation = 0.44, *p*-value = 0.007)) (Figure 1E). In addition, we measured low byproduct (C-to-A/G) editing within the editing window, overall indicative of a very high final purity of the edited cell populations (C-to-T within C-to-A/G/T: median = 99.6% and s.d. = 0.9; C-to-T within C-to-A/G/T + Indels: median = 99.5% and s.d. = 1.1, Figure 1F; Supplementary Figure S1D).

### Definition of the optimal base editing window

**FIGURE 2:**
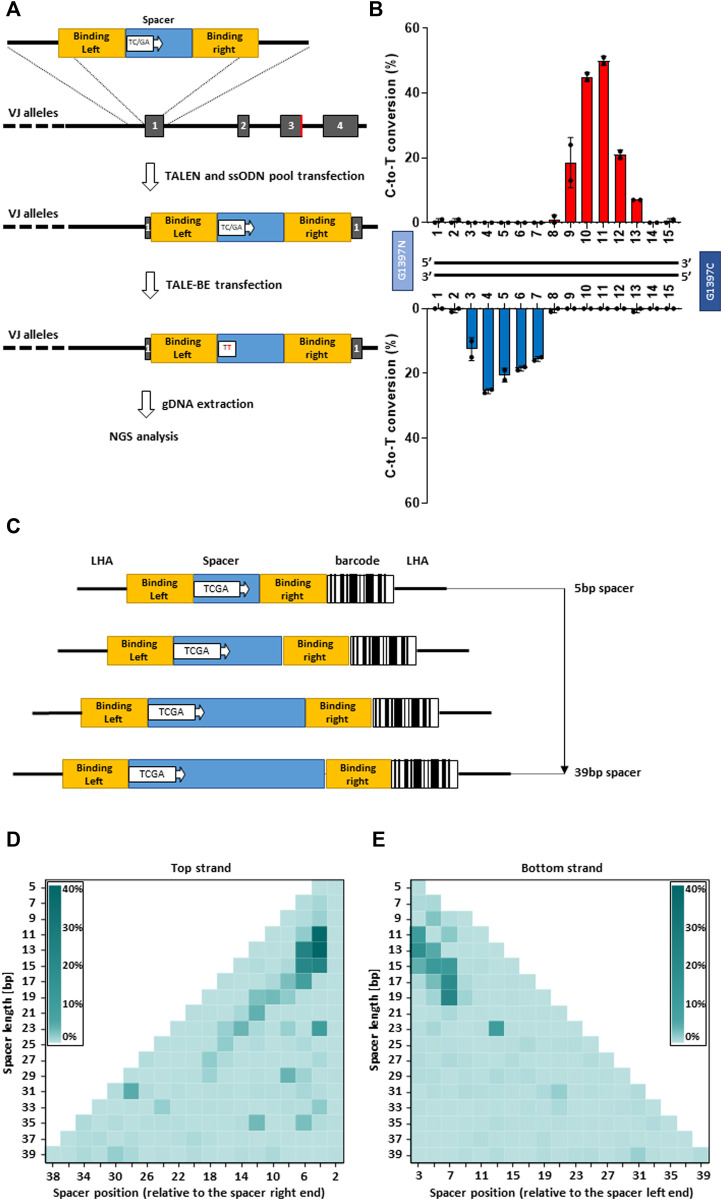
**(A)**. Scheme of the strategy to generate artificial base editor target sites. In a first step a pool of ssODN encoding various base editor spacer sequences is inserted into TRAC locus. In a second step the TALE-BE is transfected. Two days post transfection the genomic DNA is collected, and the inserted sequence is analyzed by NGS. **(B)**. Mean C-to-T conversion frequencies of the target Cs (top) or Gs (bottom) at different positions within the 15 bp TALE-BE spacer. **(C)**. Schematic representation of the ssODN pool collection with spacer length ranging from 5 to 39 bp. **(D)**. heatmap of C-to-T conversion when the TC was present on the top strand in function of the spacer length. **(E)**. heatmap of C-to-T conversion when the TC was present on the bottom strand in function of the spacer length. For all panels: *N* = 2, independent T-cells donors.

To comprehensively investigate DddA-derived cytosine base editors (TALE-BE), we designed an experimental setup allowing us to screen, in a defined genomic context, for base editing efficiency in a medium to high throughput format. We, and others, have previously demonstrated the possibility to precisely insert *via* homologous recombination, a short (50–200 bp) sequence, into the genome of primary T cells using a single strand oligonucleotide (ssODN) as a template (Roth et al., 2018; Yang et al., 2021). We decided to generate a pool of cells, containing predefined TALE-BE target sequences precisely inserted into a chosen genomic locus in the *TRAC* gene (Yang et al., 2021). Each of the TALE-BE targets contained a unique TC or GA (target for the DddA deaminase) within the spacer sequence flanked by two fixed TALE binding sequences (RVD-L and RVD-R, Figure 2A). This setup allows the uniform TALE-BE binding to the artificial target sites, excluding editing variability caused by 1) different DNA binding affinities from different TALE array protein and 2) the impact of epigenomic factors, such as chromosome relaxation around the artificial BE target sites. We first designed a collection of ssODN that contain two fixed TALE array protein binding sites from one of the most active TALE-BE identified previously on endogenous genomic loci (T-25 TALE-BE, Supplementary Table S1). The two TALE binding sequences were separated by various 15 bp spacer sequences (similar length to the previous collection of TALE-BE targeting endogenous loci). To generate the pool of cells harboring the collection of BE targets, the 30 ssODN (15 TC and 15 GA, Supplementary Table S2) oligonucleotides were mixed in equal amounts and transfected in primary T-cells by electroporation simultaneously with a TALEN targeting *TRAC* (Valton et al., 2015). In the second step, 2 days post transfection of the ssODN pool, the mRNAs encoding the T-25 TALE-BE were vectorized by electroporation. The genomic DNA of transfected cells was then harvested at day 2 post TALE-BE transfection for editing analysis (Figure 2A). The NGS analysis showed that the ssODNs were efficiently and homogenously integrated at the *TRAC* locus (read number: median = 1667.5, mean = 1686.2, s.d. = 351.7, Supplementary Figure S2A). The control sample treated without TALE-BE showed low frequencies of background mutations, whereas the samples treated with TALE-BE showed detectable and reproducible levels of C-to-T conversion (Supplementary Figures S2B,C).

The analysis further highlighted editing windows comparable to those observed with the 37 TALE-BE targeting endogenous sequences (Figure 2B, Figure 1D). Indeed, when comparing the relative activity of the two data sets, we found strong overlap for the primary site of editing at the TC of the top strand of the editing window (Pearson corr. coeff. = 0.82, Figure S2D). The overlap was less robust, but still apparent, for the secondary site of editing at the TC of the bottom strand (Pearson corr. coeff. = 0.69). Altogether, the similarity of the two datasets further validate this artificial pooled spacer approach and that the positional rules for editing are not locus dependent.

As the artificial pooled spacer approach demonstrated the possibility to dissect TALE-BE editing profiles, we expanded our ssODN collection to spacers with odd number lengths, spanning from 5 to 39 bp (i.e. 5, 7, 9, 11 … 37, 39 bp). To reduce the number of ssODN needed for the collection, a TCGA quadruplex target sequence was incorporated in the spacer at every other position (Figure 2C). This design, containing 191 unique ssODNs (Supplementary Table S3), allowed us to simultaneously interrogate editing efficiencies on both strands with a single ssODN. Additionally, to facilitate the sequence analysis, a unique barcode was added to each construct (Figure 2C). Upon filtering the NGS data to remove the reads in which the barcode conflicted with the spacer sequence, we obtained a high and homogenous representation of each ssODN (read number: median = 545, mean = 3522.6, s.d. = 7122.5, Supplementary Figure S3A). As with the previous collection (15 bp spacer), low frequencies of mutations were observed without the TALE-BE, while C-to-T conversion was robustly measured with the TALE-BE, either on the plus or minus strand (Supplementary Figure S3B). Analysis of the data indicated that, for our TALE-BE scaffold, a spacer length ranging from 11 to 17 bp to achieve optimal editing, with a 4–7 bp editing windows on the different spacers (Figure 2D, Figure 2E).

### Efficient generation of CD52 gene knock out with TALE-BE and multiplexed cell engineering

In the context of allogeneic CAR-T therapies, *CD52* can be knocked out, in combination with *TRAC*, *via* TALEN-gene editing to create resistance to alemtuzumab, a CD52-targeting monoclonal antibody used in lymphodepleting regimens (Poirot et al., 2015; Qasim et al., 2017). In order to improve the outcome of simultaneous multiplex gene knock-out, and prevent possible translocations arising with the contemporaneous use of multiple nucleases (Poirot et al., 2015), we sought to develop two base editing approaches, targeting either a splice site or the signal sequence, to promote efficient CD52 knock-outs.

**FIGURE 3:**
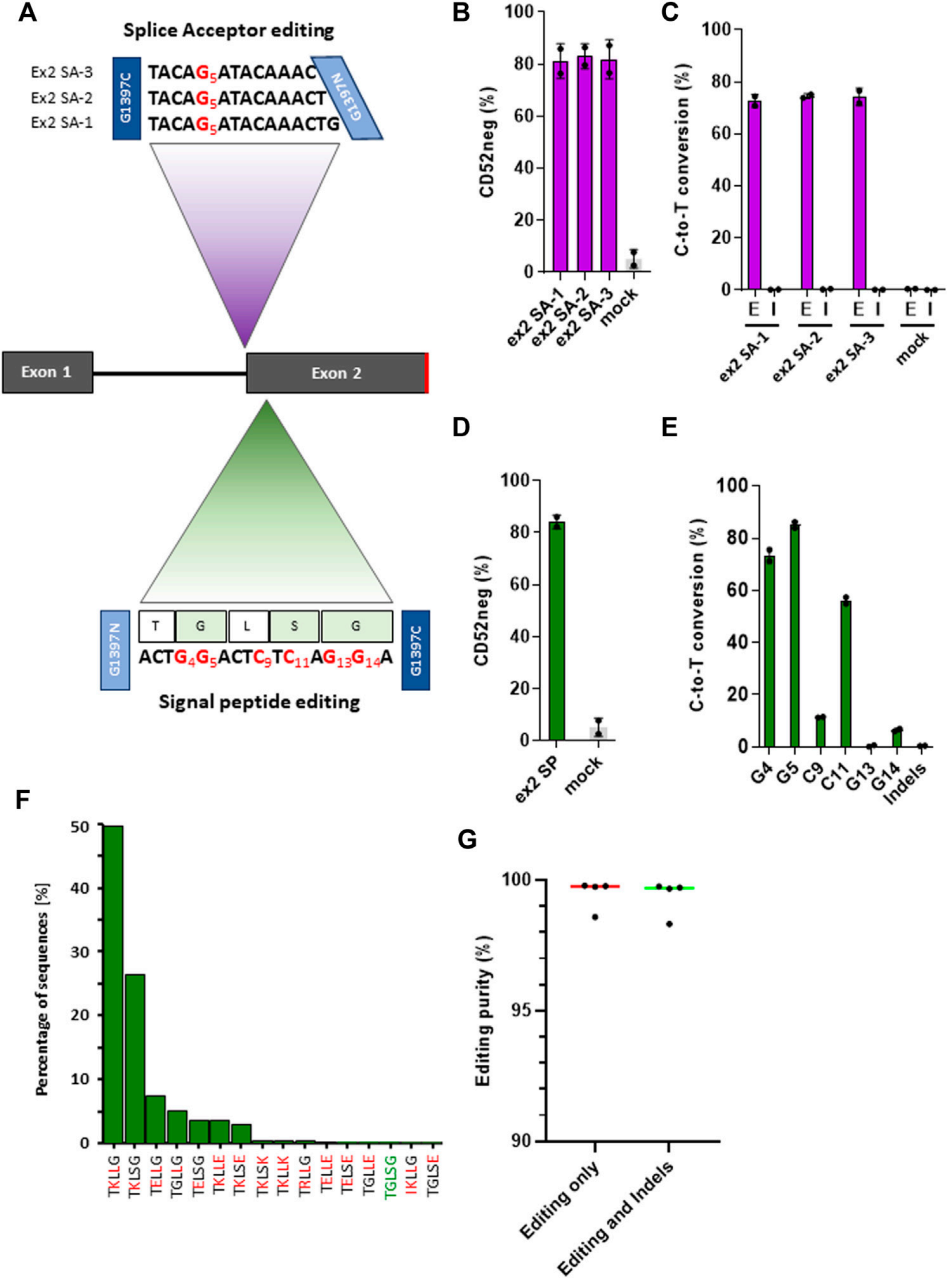
**(A)**. Schematic representation of target spacer sequence for TALE-BE targeting CD52. Top: TALE-BE (ex2 SA-1, two and 3) designed to edit CD52 exon2 splice acceptor site. The conserved G of the splice site, targeted by the TALE-BE) is depicted in red. Bottom: TALE-BE targeting CD52 signal peptide sequence in exon 2 (SP). TALE-BE spacer sequence (with targetable Cs or Gs numbered) and peptide sequence are depicted. **(B)**. CD52 negative cell frequency in the TALE-BE treated (targeting CD52 exon2 splice acceptor site) or mock electroporated PBMC populations, 6 days post electroporation, measured by flow cytometry. **(C)**. Editing **(E)** frequencies (C-to-T conversion) of the conserved G of the exon two acceptor splice site (Editing) and the indel **(I)** frequencies within the target locus, measured by NGS 6 days post transfection. **(D)**. CD52 negative cell frequency in the TALE-BE treated (targeting CD52 signal sequence in exon2) or mock electroporated PBMC populations, 6 days post electroporation, measured by flow cytometry. **(E)**. Editing frequencies (C-to-T conversion) at different position within the TALE-BE target spacer (CD52 signal sequence in exon2) and indel frequencies within the target locus, measured by NGS at Day 6 post transfection. **(F)**. Frequencies of peptide species created by the TALE-BE targeting the CD52 signal sequence in exon 2. The first 16 most abundant species are presented. Mutation relative to the native signal peptide are in red. (*N* = 2, independent T-cells donors). **(G).** Editing purity within the cell population. Aggregate of the four TALE-BE targeting CD52. Left: C-to-T conversion within the C-to-A/G/T population. Right: C-to-T conversion within the C-to-A/G/T + Indels population. For all panels: *N* = 2, independent T-cells donors.

Because the CD52 gene only has two exons, and the exon two contains the sequence coding for the mature peptide, splice site mutations at the intron 1/exon 2 junction conserved motif would presumably disrupt RNA splicing, causing the retention of intron 1 and ultimately leading to the loss of CD52 (Figure 3A). Taking into consideration the sequences surrounding the target splice site and some constraints in the TALE-BE design: T at the extremities of the target, 16 bp TALE binding sequences, 11–17 bp spacer length, we were able to identify 34 potential base editors targeting the conserved G within the splice site. We then narrowed down the BE candidate list, according to the TC positioning data we gathered from the artificial pooled spacer experiments, to three lead BEs (Figure 3A). Primary T cells were transfected with mRNA encoding these three pairs of TALE-BE. Seven days post transfection, phenotypic CD52 knock-out was monitored by flow cytometry and splice site editing was measured by NGS. We observed high level of phenotypic knock-out for the three TALE-BE (Figure 3B; Supplementary Figure S4, ex2 SA-1 mean 81.1% ± 4.7%, ex2 SA-2 mean 83% ± 3.4% and ex2 SA-3 mean 81.9%, ± 5.3%), correlating with editing levels (ex2 SA-1 mean 72.6%, ± 1.7%, ex2 SA-2 mean 74.5%, ± 0.6%. and ex2 SA-3 mean 74.2%, ± 2.3%, Figure 3C). As expected from our previous datasets, NGS data analysis results showed very low levels of Indels at these sites (ex2 SA-1 mean 0.16%, ± 0.05%; ex2 SA-2 mean 0.28%, ± 0.06%; ex2 SA-3 mean 0.12%, ± 0.02%, Mock transfected mean 0.01%, ± 0.005%; Figure 3C).

Mutations in signal peptides has been shown to disrupt the processing and the translocation of nascent peptides and thus impair the surface expression of certain genes (Wiren et al., 1989). We thus evaluated a second CD52 KO approach by designing a TALE-BE targeting CD52 signal sequence. This approach could potentially lead to 1) a silent mutation at Leu23 residue and 2) several amino acid changes (Gly22Lys, Ser24Leu and Gly25Lys) (Figure 3A). We anticipated that such changes in the residues, mutating a hydrophobic glycine to a highly charged lysine, and a polar serine to a hydrophobic leucine in the signal peptide, would significantly impact the ability for the signal peptide to correctly direct translocation. Indeed, 6 days post TALE-BE mRNA transfection (ex2 SP), we observed by flow cytometry an average of 84.2% ( ± 1.8%) CD52 negative cells (Figure 3D; Supplementary Figure S4). The NGS sequencing analysis revealed that all six positions were mutated, albeit at different levels (mean editing frequencies: G [4]: 73.65 ± 1%, G [5]: 85.65 ± 0.7%, C [9]: 11.4 ± 0.1% C [11]: 56.5 ± 0.9%, G [13]: 0.6 ± 0.1, G [14]:6.5 ± 0.5%) (Figure 3E). Altogether, we identified editing leading to potentially 34 different species (at the protein level), including the WT, present in different proportions (Figure 3F; Supplementary Table S4).

To evaluate possible off-target editing of the four CD52 TALE-BEs (targeting the splice site and the signal sequence), we generated *in silico* a list of potential off-site targets for these BEs, covering altogether 307 unique sites (207 experimentally investigated, Supplementary Table S5). Targeted amplicon sequencing using a multiplexed amplicon sequencing assay (Chaudhari et al., 2020) was performed and the analysis did not demonstrate evidence of editing (95 sites for ex SA-1, 72 sites for ex2 SA-2, 89 sites for ex2 SA-3 and 35 sites for ex2 SP, Supplementary Table S6, 1-2 independent T-cells donors).

Having achieved very high phenotypic knock-out (median CD52 negative population: 82.1%) and editing purity (median = 99.7 and s.d. = 0.6, Figure 3G), we further evaluated the possibility to perform multiplex gene editing using two different molecular tools, a nuclease and a base editor. As a proof of concept, we combined a TALEN targeting *TRAC* and either a TALEN or a base editor (TALE-BE ex2 SP) targeting CD52, a combination of edits reported for the generation of allogeneic CAR T-cells (Poirot et al., 2015). We detected high and similar levels of phenotypic double gene knock-out by flow cytometry in both TALEN/TALEN and TALEN/TALE-BE treated samples (79% and 75% respectively, Supplementary Figure S5). However, translocations between the two targeted loci, detected by multiplexed amplicon sequencing, (Amit et al., 2021), were only observed in the TALEN/TALEN treated sample (479 reads out of 224,406 for the TALEN/TALEN sample; 0 reads out of 144,323 for the TALEN/BE sample, N = 1, 1 single T-cell donor). Overall, we believe that such a multi-tool approach for multiplex gene editing has the potential to streamline development of products by easing QCs (absence of translocation) and more globally improve safety of multiplex cell engineering.

## Discussion

Base editing represents one of the newest gene editing technologies. Recently, the TALE scaffold was demonstrated to be compatible with the creation of a new class of DddA-derived cytosine base editors. In this study, we combined the screening of several base editors targeting various endogenous loci with the development of a simple and robust medium-throughput approach to interrogate the determinants of editing by TALE-BE. This medium-throughput screening strategy is taking advantage of the highly efficient and precise TALEN mediated ssODN knock-in in primary T cells and allowed to assess the TALE-BE editing efficiency on hundreds of different targets *in cellulo*. Because all BE artificial target sequences are inserted into the same predefined locus in the genome, this method allowed us to focus on how target/spacer sequence variations could affect TALE-BE while excluding factors such as DNA binding affinities or epigenetic variations. The experimental results pointed out an optimal 13–17 bp spacer length window for editing, with the G1397C-bearing arm of the TALE-BE being placed 4–7 bp down the 3’ direction of the target TC for the best editing activity.

Although CRISPR/Cas cytosine base editors do not introduce the intended mutations through double strand break (DSB) repair by the non-homologous end joining (NHEJ) pathway, significant levels of unwanted indels creation has often been reported (Thuronyi et al., 2019; Doman et al., 2020). Since the first report of such designer base editors, improvements of editing efficiencies were often obtained at the cost of editing purity (Indels, byproduct mutation or bystander mutations) or *vice versa* (Komor et al., 2017; Thuronyi et al., 2019; Doman et al., 2020; Tran et al., 2022). Often this is achieved by the restoring of partial function to the dead Cas9 employed to target the base editing domain to the site of interest. By restoring one of two catalytic sites (adding back the catalytic His at position 840 while retaining the inactivating Asp10Ala), researchers were able to increase editing by nicking the non-edited strand (Komor et al., 2016). While extremely precise introduction of the intended mutation (high purity of the final product) is a prerequisite for application such as gene correction, bystander and byproduct edits might be of a lesser concern for gene disruption applications. However, generation of DSBs by base editors may raise greater concerns as CRISPR/Cas base nucleases have been recently associated with major on-target genome instability or chromosomal abnormalities (Weisheit et al., 2020; Alanis-Lobato et al., 2021; Boutin et al., 2021; Papathanasiou et al., 2021; Boutin et al., 2022; Geng et al., 2022; Nahmad et al., 2022; Sánchez-Rivera et al., 2022). In this study we only found marginal byproduct mutation (C-to-A/G), and more importantly low Indel creation, by TALE-BE looking at dozens of these molecular tools, even at high editing frequencies (>80% in bulk population), occurring at levels similar to, or lower than, those reported by other groups (Mok et al., 2020; Lim et al., 2022). One possibility for these low levels of Indels might be the result of a lack of reliance on opposite strand nicking for improved editing by TALE-BE. By avoiding the need for a nick on one strand opposite an edited abasic site on the other, these editors may simply do less potentially DSB-causing damage at the target site. Additional studies will need to be carried out to further elucidate whether these are the resulting byproducts of an imperfect DNA repair pathway, or perhaps the results of rare double strand breaks occurring during the replication process. Furthermore, a careful design of the BE positioning, would allow to prevent or minimize bystander mutations.

Base editors have been used to edit or mutate conserved genetic elements such as enhancers (Zeng et al., 2020), start codons (Wang et al., 2020), splice sites (Kluesner et al., 2021), branch points (Yuan et al., 2018), and conserved active sites (Hanna et al., 2021). We estimated that ∼46,000 (46,608) splice sites in the genome could potentially be targeted by TALE-BE, impacting 15,279 different transcripts, representing 76.57% of all the transcripts in human genome (Frankish et al., 2021) and, overall, indicating that splice site editing could be a viable approach for gene knock-out by TALE-BE. To demonstrate the feasibility of such an approach, we designed highly efficient TALE-BE targeting the conserved G of the intron 1/exon 2 junction splice site of the CD52 gene. We further demonstrated that as an alternative to splice site editing, targeting the signal peptide can also lead to efficient surface protein knock-out.

Base editors represent promising molecular tools for multiplex gene engineering, though currently limited to knock-out or gene corrections. Here we demonstrated the feasibility of efficient multiplex gene engineering using a combination of two different molecular tools, a nuclease, and a base editor. Such a multiplex/multitool strategy presents several advantages. First, it prevents creation of translocations often observed with the simultaneous use of several (>1) nucleases (Poirot et al., 2015; Webber et al., 2019; Samuelson et al., 2021). And second, it allows the possibility to go beyond multiple knock-outs while still allowing gene knock-in at the nuclease target site, altogether extending the scope of possible application, while better controlling the engineered cell population outcome (e.g., absence of translocations). The precise positional rules we have determined for TALE-BE, low frequency of unwanted indels generated, and the increased accessibility to additional cell compartments allowing access beyond the traditional nuclear targets, expands the potential scope of such a TALE-based multiplex/multitool strategy beyond the capabilities of most other non-TALE editing tools.

## Materials and methods

### T cell culture

Cryopreserved human PBMCs were acquired from ALLCELLS. PBMCs were cultured in X-vivo-15 media (Lonza Group), containing 20 ng/ml human IL-2 (Miltenyi Biotec), and 5% human serum AB (Seralab). Human T cell activator TransAct (Miltenyi Biotec) was used to activate T cells at 25 µL TransAct per million CD3^+^ cells the day after thawing the PBMCs. TransAct was kept in the culture media for 72 h.

### Small scale mRNA production

Plasmid of the 37 TALE-BE and 37 matching TALEN, containing a T7 promoter and a polyA sequence, were produced as non-clonal after assembly (transformant was directly inoculated for culture and plasmid preparation). The plasmids were then linearized with SapI (NEB) and mRNA was produced by *in vitro* transcription (NEB HiScribe ARCA, NEB).

### Small scall TALEN and TALE-BE testing (37 endogenous targets and TRAC/CD52 multiplex engineering)

T cells activated with TransAct (Miltenyi Biotec) for 3 days were passaged into fresh complete media containing 20 ng/ml human IL-2 (Miltenyi Biotec), and 5% human serum AB (Seralab) 10–12 h s before transfection.

The harvested cells were washed once with warm PBS. 1E6 PBS washed cells were pelleted and resuspended in 20 µL Lonza P3 primary cell buffer (Lonza). 1 μg/arm/million cells of mRNA for TALEN or TALE-BE was mixed with the cells and then the cell mixture was electroporated using the Lonza 4D-Nucleofector under the EO115 program for stimulated human T cells. After electroporation, 80 µL warm complete media was added to the cuvette to dilute the electroporation buffer, the mixture was then carefully transferred to 400 ml pre-warmed complete media in 48-well plates. TALEN transfected cells were incubated at 30°C for an overnight culture and then transferred back to 37°C incubator. TALE-BE transfected cells were incubated at 37°C throughout the process. Cells were harvested at Day 6 post transfection for gDNA extraction and NGS analysis.

### Large scale TALEN and TALE-BE mRNA production (CD52 targeting BEs)

Plasmids encoding the TRAC TALEN contained a T7 promoter and a polyA sequence. The TALEN mRNA from the TRAC TALEN plasmid was produced by Trilink. Sequence targeted by the TRAC TALEN (17-bp recognition sites, upper case letters, separated by a 15-bp spacer): (TTC​CTC​CTA​CTC​ACC​ATc​agc​ctc​ctg​gtt​atG​GTA​CAG​GTA​AGA​GCA​A).

The TALEN mRNA from the CD52 TALEN plasmid was produced by Trilink. Sequence targeted by the CD52 TALEN (17-bp recognition sites, upper case letters, separated by a 15-bp spacer): (TTC​CTC​CTA​CTC​ACC​ATc​agc​ctc​ctg​gtt​atG​GTA​CAG​GTA​AGA​GCA​ACG​CCT​GGC​A).

Plasmids encoding TALE-BE T-25 and CD52 TALE-BE contained a T7 promoter and a polyA sequence (TALE-BE Sequence and target sequence in Supplementary info). Sequence verified plasmids were linearized with SapI (NEB) befor *in vitro* mRNA synthesis. mRNA was produced with NEB HiScribe™ T7 Quick High Yield RNA Synthesis Kit (NEB). The 5′capping reaction was performed with ScriptCap™ m7G Capping System (Cellscript). Antarctic Phosphatase (NEB) was used to treat the capped mRNA and the final cleanups was performed with Mag-Bind TotalPure NGS beads (Omega bio-tek) and Invitrogen DynaMag-2 Magnet (ThermoFisher).

### ssODN repair template transfection

The ssODN pool targeting the TRAC locus (Supplementary Table S2, S3) were ordered from Integrated DNA Technologies (IDT) and resuspended in ddH2O at 50 pmol/μl.

T cells activated with TransACT for 3 days were passaged into fresh complete media containing 20 ng/ml human IL-2 (Miltenyi Biotec), and 5% human serum AB (Seralab) 10–12 h s before transfection.

The harvested cells were washed once with warm PBS. 1E6 PBS washed cells were pelleted and resuspended in 20 µl Lonza P3 primary cell buffer (Lonza). 200 pmol ssODN pool and 1μg/arm of TRAC TALEN were mixed with the cell and then the cell mixture was electroporated using the Lonza 4D-Nucleofector under the EO115 program for stimulated human T cells. After electroporation, 80 µl warm complete media was added to the cuvette to dilute the electroporation buffer, the mixture was then carefully transferred to 400 ml pre-warmed complete media in 48-well plates. Cells transfected with ssODN and TALEN were then incubated at 30°C until 24 h s post TALEN transfection before transfer back to 37°C.

Cells with ssODN KI were cultured for 2 days before harvesting for TALE-BE treatment. The harvested cells were washed once with warm PBS. 1E6 PBS washed cells were pelleted and resuspended in 20 µL Lonza P3 primary cell buffer (Lonza). 1μg/arm of TALE-BE T-25 were mixed with the cell and then the cell mixture was electroporated using the Lonza 4D-Nucleofector under the EO115 program for stimulated human T cells. After electroporation, 80 µL warm complete media was added to the cuvette to dilute the electroporation buffer, the mixture was then carefully transferred to 400 ml pre-warmed complete media in 48-well plates. Cells transfected with TALE-BE incubated at 37°C for two more days before harvesting for gDNA extraction and NGS analysis.

### Large scale CD52 TALE-BE testing

T cells activated with TransACT for 3 days were passaged into fresh complete media containing 20 ng/ml human IL-2 (Miltenyi Biotec), and 5% human serum AB (Seralab) 10–12 h s before transfection.

The harvested cells were washed twice with Cytoporation Media T (BTXpress, 47-0002). 5E6 washed cells were pelleted and resuspended in 180 µL Cytoporation Media T. 2μg/arm/million cells of TALE-BE mRNA was mixed with the cells to a final volume of 200 μL and then the cell/mRNA mixture was electroporated using the BTX Pulse Agile in 0.4 cm gap cuvettes. After electroporation, 180 µL warm complete media was added to the cuvette to dilute the electroporation buffer, and the mixture was then carefully transferred to 2 ml pre-warmed complete media in 12-well plates. TALE-BE transfected cells were incubated at 37°C throughout the process. Cells were harvested at Day 6 post transfection for gDNA extraction and NGS analysis.

### Genomic DNA extraction

Cells were harvested and washed once with PBS. Genomic DNA extraction was performed using Mag-Bind Blood & Tissue DNA HDQ kits (Omega Bio-Tek) following the manufacturer’s instructions.

### Targeted PCR and NGS

100 μg genomic DNA was used per reaction in a 50 μL reaction with Phusion High-Fidelity PCR Master Mix (NEB). The PCR condition was set to 1 cycle of 30 s at 98°C; 30 cycles of 10 s at 98°C, 30s at 60°C, 30 s at 72°C; 1 cycle of 5 min at 72°C; hold at 4°C. The PCR product was then purified with Omega NGS beads (1:1.2 ratio) and eluted into 30 μL of 10 mM Tris buffer pH7.4. The second PCR which incorporates NGS indices was then performed on the purified product from the first PCR. 15 ul of the first PCR product were set in a 50 μL reaction with Phusion High-Fidelity PCR Master Mix (NEB). The PCR condition was set to 1 cycle of 30 s at 98°C; 8 cycles of 10 s at 98°C, 30 s at 62°C, 30s at 72°C; 1 cycle of 5 min at 72°C; hold at 4°C. Purified PCR products were sequenced on MiSeq (Illumina) on a 2 × 250 nano V2 cartridge.

## Flow cytometry

TRAC KO was monitored using an anti-TCRa/b antibody (Biolegend, #306732, clone IP26, BV605). CD52 KO was monitored using an anti-52 antibody (BD Biosciences, #563609, Clone 4C8, AlexaFlour488). Flow cytometry was performed on BD FACSCanto (BD Biosciences) and data analysis processed with FlowJo. Cell population was first gated for lymphocytes (SSC-A vs. FSC-A) and singlets (FSC-H vs. FSC-A). The lymphocyte gate was further analyzed for expression of CD52 and -TCRa/b expression from this gated population.

### *In Silico* off-site prediction

To evaluate possible off-target editing of the CD52 TALE-BEs, we generated *in silico* a list of potential off site targets of these BEs. That list was generated as follow. The TALE-BE have two binding sequences of 17 bp separated by a spacer. These binding sequences begin necessarily by a T. Hence, we first selected as potential targets all genomic sequences starting with a T, ending with an A, and having a size comprised between 27 bp and 67 bp (both included), allowing for spacers ranging from 10 to 40 bp. Then, the number of mismatches between the binding sequences of the potential target versus the actual TALE-BE target was counted. If that total number was greater than 8, the potential target was removed. Finally, all potential targets lacking a G in the left half of the spacer, or a C in the right half of the spacer (editing windows) were discarded.

### Off-site and translocation multiplexed amplicon sequencing

rhAmp primers were designed on the on-target and/or off-target sites established by an *in silico* off-site prediction. Locus-specific forward and reverse primers were obtained from Integrated DNA Technologies (IDT) either in ready to use pools or individually plated, and use accordingly to IDT protocol for RNase H2-dependent multiplex assay amplification (1 cycle of s at 95°C 10 min; 14 cycles of 15s at 95°C followed by 8 min at 65°C; 1 cycle of 15 min at 99.5°C; hold at 4°C) followed by a universal PCR to add indexes (i5 or i7) for NGS (1 cycle of s at 95°C 3 min; 24 cycles of 15 s at 95°C followed by 30 s at 60°C and 30s at 72°C; 1 cycle of 1 min at 72°C; hold at 4°C). Purified PCR amplicons were sequenced on a NextSeq (Illumina) on a NextSeq 500/550 Mid Output Kit (150 cycles) cartridge.

### Off-site and translocation multiplexed amplicon analysis

rhAmp sequencing reads were retrieved, trimmed for quality and aligned against the human genome (assembly GRCh38). Then, sequences that were aligned at positions corresponding to the generated potential off-sites were retrieved, if more than 200 reads were aligned. From these sequences, C>T and G>A and unedited C and G were counted. A χ^2^ test of independent variables between the control and treated samples was performed. Sites with a *p*-value less than 5% were selected.

## Data Availability

The original contributions presented in the study are included in the article/Supplementary Material, further inquiries can be directed to the corresponding author.
