# Supplementary material for: Efficient multitool/multiplex gene engineering with TALE-BE
Source: Front Bioeng Biotechnol. 2022 Nov 10;10:1033669. doi: 10.3389/fbioe.2022.1033669 (PMC9684181; doi:10.3389/fbioe.2022.1033669)
Supplement: Supplementary file 3 [file Presentation1.pptx]

## Slide 1
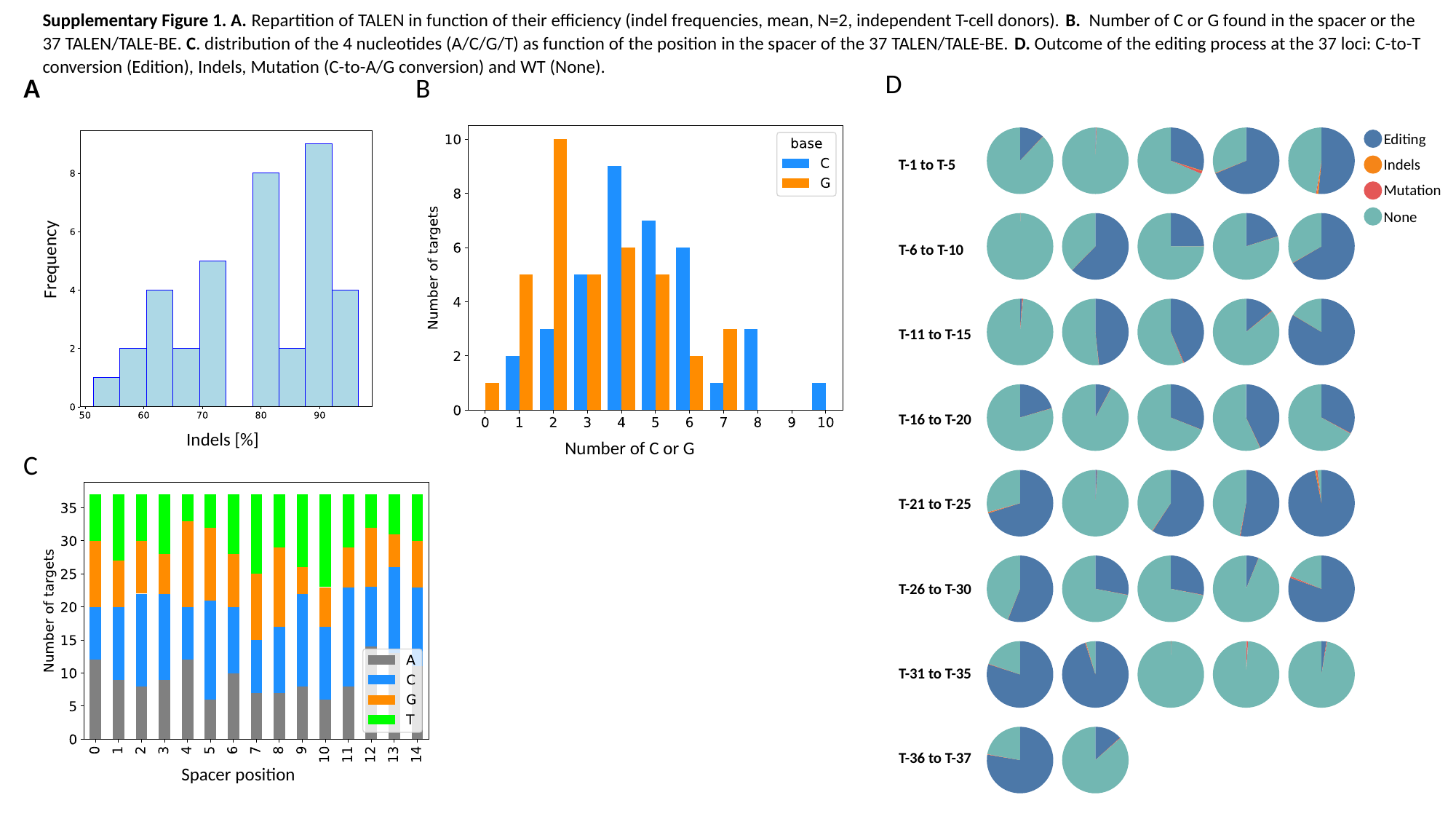

Supplementary Figure 1. A. Repartition of TALEN in function of their efficiency (indel frequencies, mean, N=2, independent T-cell donors). B. Number of C or G found in the spacer or the 37 TALEN/TALE-BE. C. distribution of the 4 nucleotides (A/C/G/T) as function of the position in the spacer of the 37 TALEN/TALE-BE. D. Outcome of the editing process at the 37 loci: C-to-T conversion (Edition), Indels, Mutation (C-to-A/G conversion) and WT (None).
D
A
B
Editing
Indels
Mutation
None
T-1 to T-5
Frequency
T-6 to T-10
T-11 to T-15
T-16 to T-20
Indels [%]
Number of C or G
C
T-21 to T-25
T-26 to T-30
T-31 to T-35
T-36 to T-37
Spacer position

## Slide 2
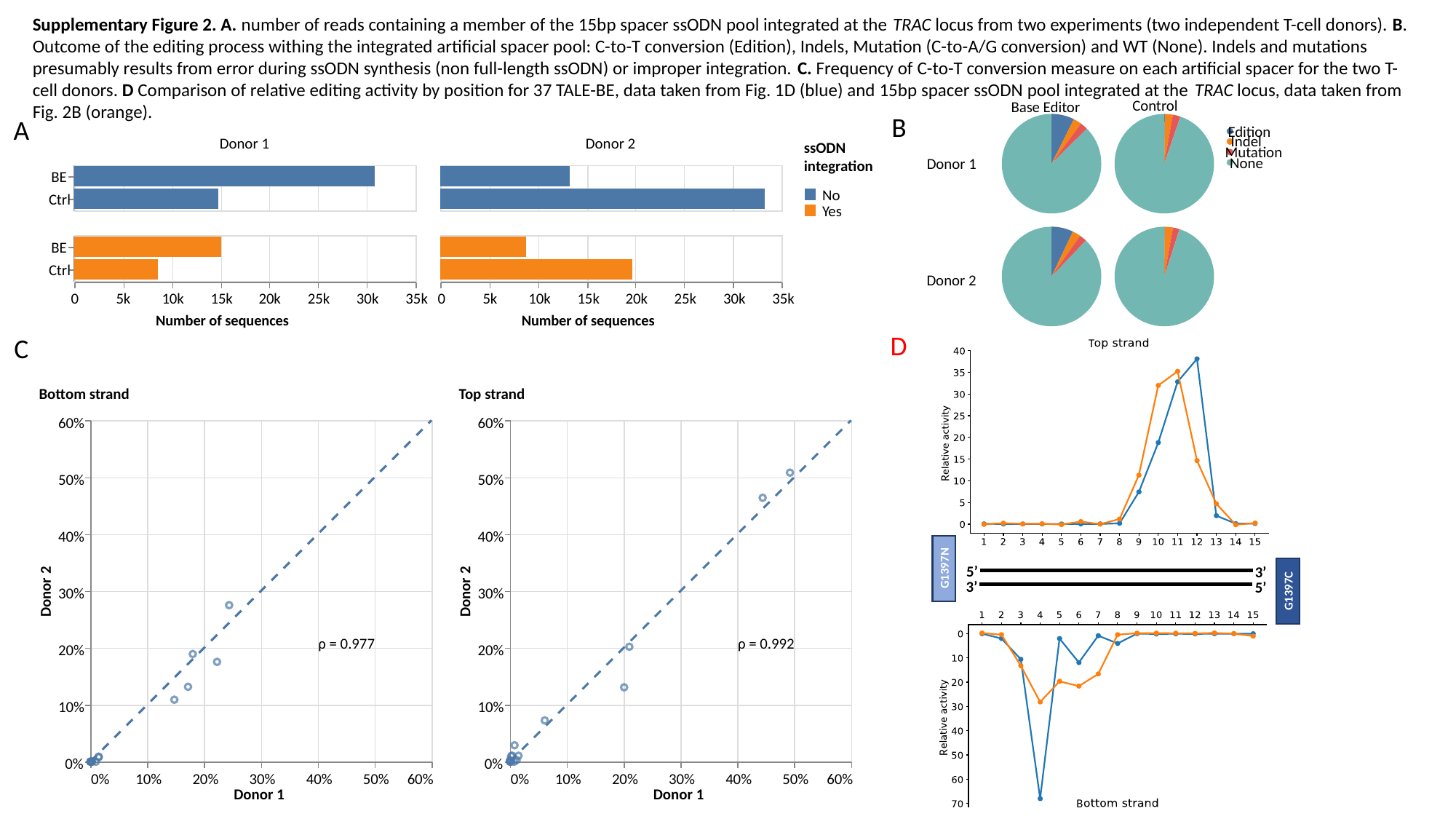

Supplementary Figure 2. A. number of reads containing a member of the 15bp spacer ssODN pool integrated at the TRAC locus from two experiments (two independent T-cell donors). B. Outcome of the editing process withing the integrated artificial spacer pool: C-to-T conversion (Edition), Indels, Mutation (C-to-A/G conversion) and WT (None). Indels and mutations presumably results from error during ssODN synthesis (non full-length ssODN) or improper integration. C. Frequency of C-to-T conversion measure on each artificial spacer for the two T-cell donors. D Comparison of relative editing activity by position for 37 TALE-BE, data taken from Fig. 1D (blue) and 15bp spacer ssODN pool integrated at the TRAC locus, data taken from Fig. 2B (orange).
Control
Base Editor
Donor 1
Donor 2
Edition
Indel
Mutation
None
B
A
Donor 1
Donor 2
ssODN
integration
No
Yes
BE
Ctrl
BE
Ctrl
0
5k
10k
15k
20k
25k
30k
35k
0
5k
10k
15k
20k
25k
30k
35k
Number of sequences
Number of sequences
D
C
T
R
5’
G1397N
3’
3’
5’
G1397C
R
B
Bottom strand
60%
50%
40%
30%
20%
10%
0%
Donor 2
ρ = 0.977
0%
10%
20%
30%
40%
50%
60%
Donor 1
Top strand
60%
50%
40%
30%
20%
10%
0%
Donor 2
ρ = 0.992
0%
10%
20%
30%
40%
50%
60%
Donor 1

## Slide 3
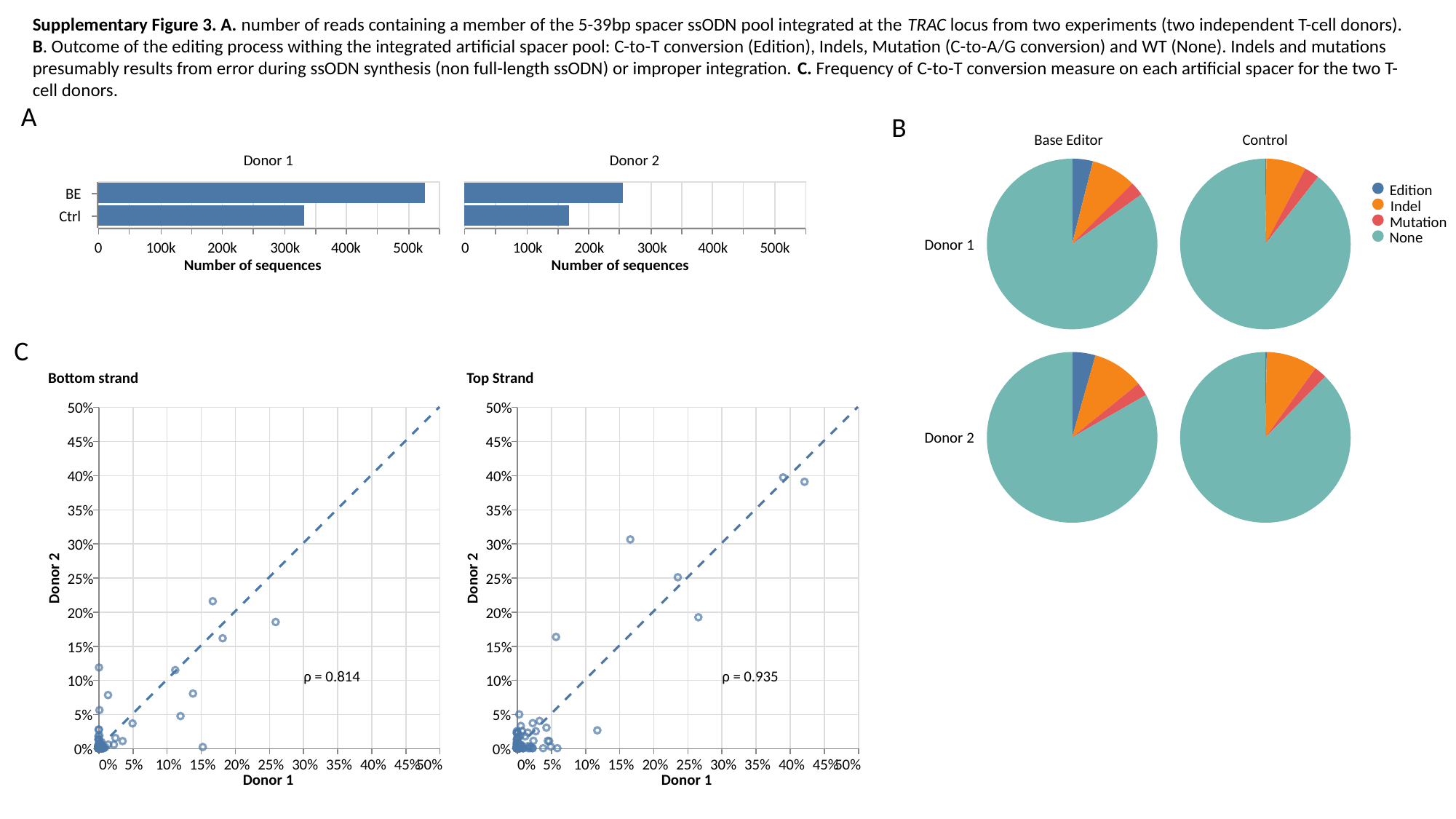

Supplementary Figure 3. A. number of reads containing a member of the 5-39bp spacer ssODN pool integrated at the TRAC locus from two experiments (two independent T-cell donors). B. Outcome of the editing process withing the integrated artificial spacer pool: C-to-T conversion (Edition), Indels, Mutation (C-to-A/G conversion) and WT (None). Indels and mutations presumably results from error during ssODN synthesis (non full-length ssODN) or improper integration. C. Frequency of C-to-T conversion measure on each artificial spacer for the two T-cell donors.
A
B
Base Editor
Control
Donor 1
Donor 2
Edition
Indel
Mutation
None
Donor 1
Donor 2
BE
Ctrl
0
100k
200k
300k
400k
500k
0
100k
200k
300k
400k
500k
Number of sequences
Number of sequences
C
Bottom strand
50%
45%
40%
35%
30%
25%
20%
15%
10%
5%
0%
Donor 2
ρ = 0.814
0%
5%
10%
15%
20%
25%
30%
35%
40%
45%
50%
Donor 1
Top Strand
50%
45%
40%
35%
30%
25%
20%
15%
10%
5%
0%
Donor 2
ρ = 0.935
0%
5%
10%
15%
20%
25%
30%
35%
40%
45%
50%
Donor 1

## Slide 4
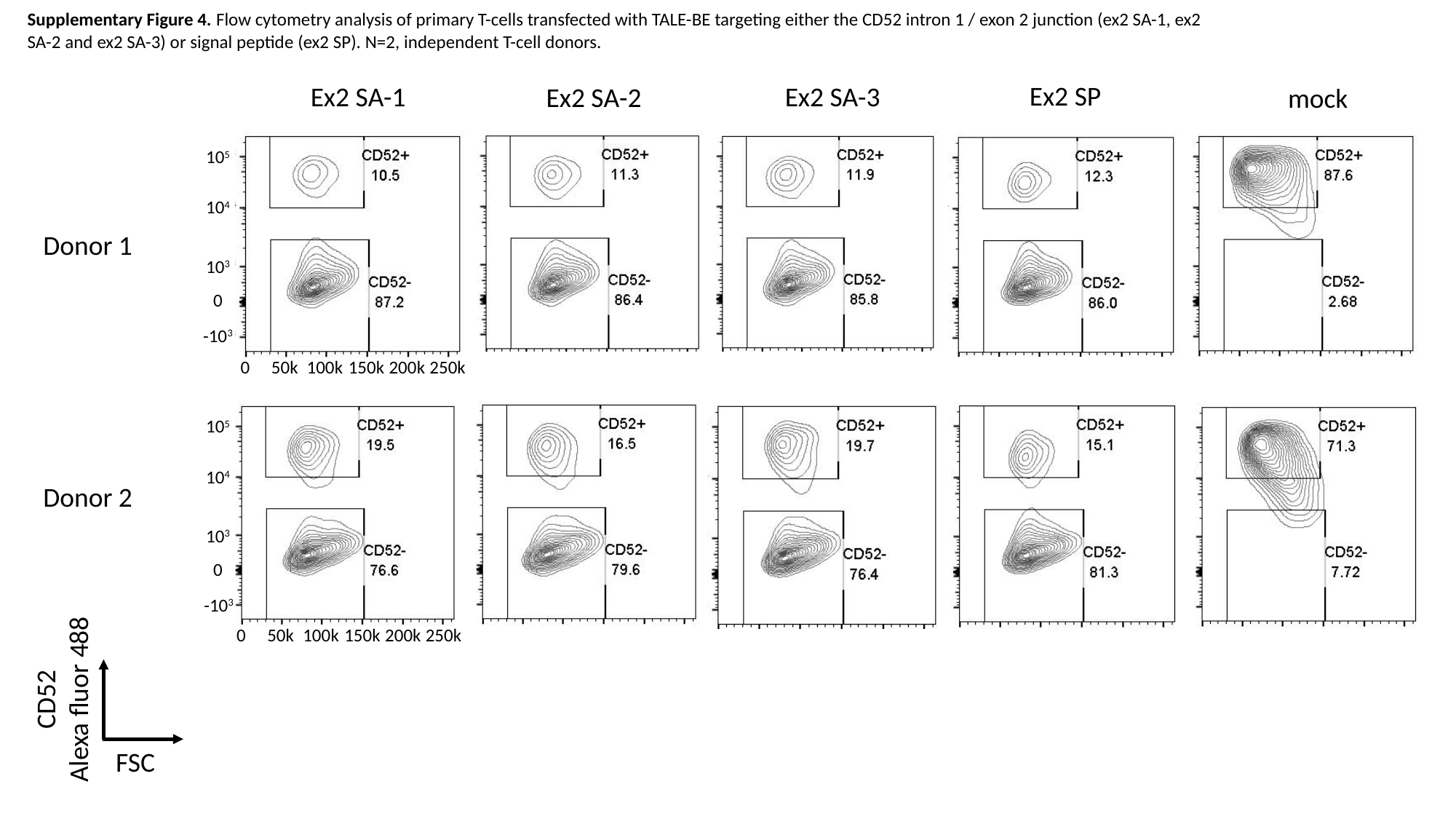

Supplementary Figure 4. Flow cytometry analysis of primary T-cells transfected with TALE-BE targeting either the CD52 intron 1 / exon 2 junction (ex2 SA-1, ex2 SA-2 and ex2 SA-3) or signal peptide (ex2 SP). N=2, independent T-cell donors.
Ex2 SP
Ex2 SA-1
Ex2 SA-3
Ex2 SA-2
mock
105
104
Donor 1
103
0
-103
0
50k
100k
150k
200k
250k
105
104
Donor 2
103
0
-103
CD52
Alexa fluor 488
FSC
0
50k
100k
150k
200k
250k

## Slide 5
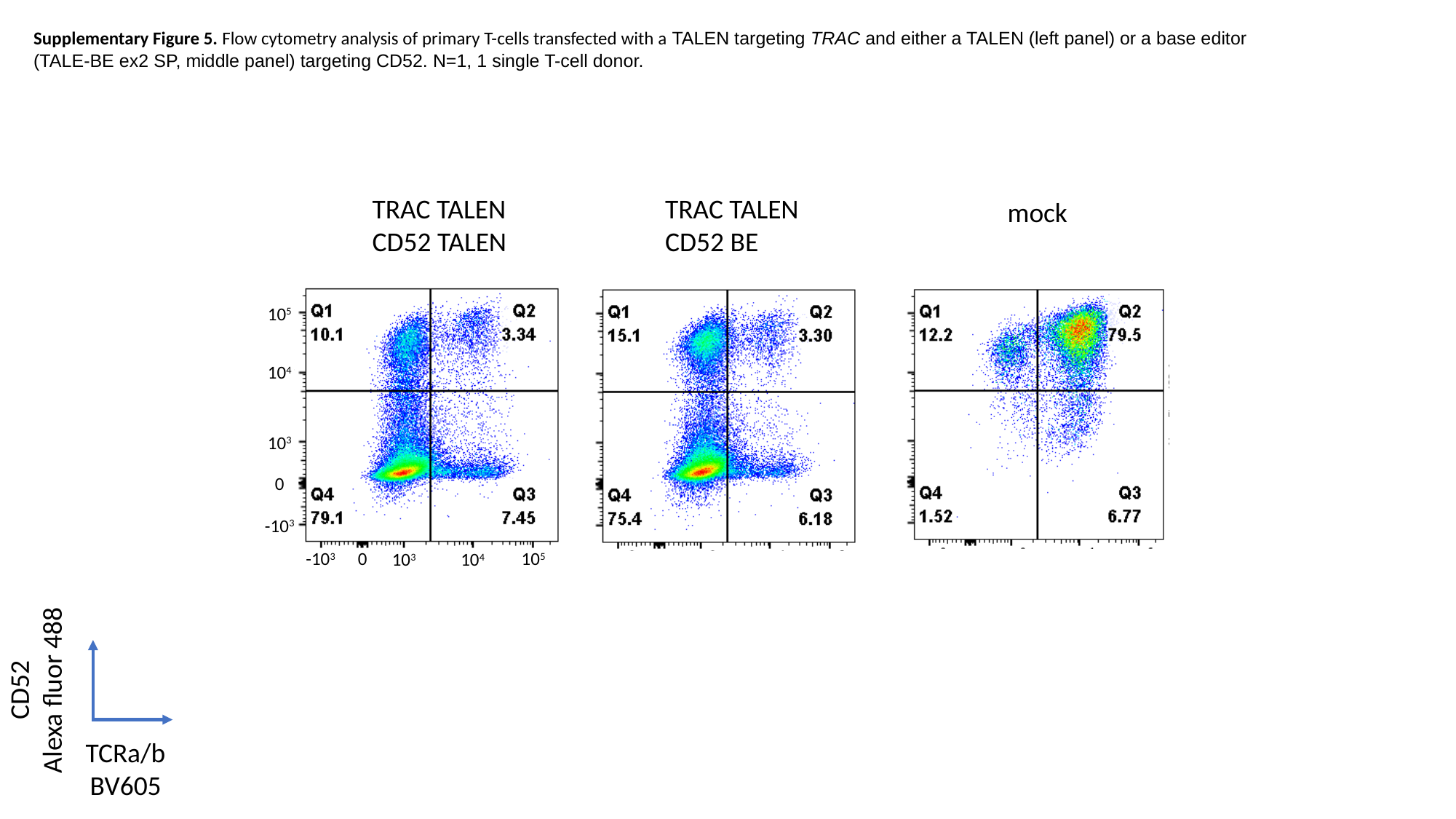

Supplementary Figure 5. Flow cytometry analysis of primary T-cells transfected with a TALEN targeting TRAC and either a TALEN (left panel) or a base editor (TALE-BE ex2 SP, middle panel) targeting CD52. N=1, 1 single T-cell donor.
TRAC TALEN
CD52 BE
TRAC TALEN
CD52 TALEN
mock
105
104
103
0
-103
0
105
-103
104
103
CD52
Alexa fluor 488
TCRa/b
BV605
